# Quantitative paradigm for analysis of multiple subtypes of immune system cells in lung cancer tissues

**DOI:** 10.1186/2051-1426-2-S3-P140

**Published:** 2014-11-06

**Authors:** Mirza Peljto, Justin Major, Joseph S Krueger, Holger Lange, Famke Aeffner, G David Young, JD Alvarez, Michael Sharp, Manuel Alejandro Sepulveda, AJ Milici

**Affiliations:** 1 Flagship Biosciences, Boulder, CO, USA; 2 Janssen R & D, Spring House, PA, USA

## 

The state of the immune system is reflected, in part, by the cell populations present in individual tissues which reflect the tumor microenvironment (TME). Several studies have suggested that understanding the TME constituents is useful for predicting drug response outcomes. Despite the biological significance of various inflammatory cell types in the TME, a widely accepted quantitative paradigm that allows the comparison of multiple subtypes of inflammatory cells in tissues is lacking. This is largely due to our inability to integrate key spatial information for multiple biomarkers across whole tissue sections, understand the concordance of individual biomarkers/cell types, account for heterogeneity in the region of analysis, and the absence of validated correlative scoring paradigms to measure against drug response. Finally, if these hurdles of understanding are met, a system needs to be created which can efficiently and reproducibly asses these readouts in a clinical-use environment. In this study, we revealed by immunohistochemistry distinct populations of immune system cells on serial sections of clinical lung cancer tissues. The immune cell markers evaluated included CD68, CD33, CD11b, FoxP3, CD4, and CD8. Whole tissue image analysis (tIA) was performed using Flagship's CellMap™ algorithm tool for each biomarker. Biomarker expression was analyzed on cell by cell basis for the tumor microenvironment and tabulated across the whole tissues. In order to integrate spatial information of CD68, CD33, CD11b, FoxP3, CD4, and CD8 biomarker expression from each slide onto a single tissue section, we utilized Flagship's patented FACTS™ tIA tool. This tool allows for an overlay of biomarker content information on to a single reference slide (H&E), and thus can provide key spatial information on biomarker expression relationships and presence of distinct cell populations in relation to each other. These studies directly demonstrate an effective utilization of combination of IHC with a set of Flagship tIA-based tools to score and analyze immune cell biomarker content in whole clinical tissue samples. Importantly, these tools can spatially correlate multiple biomarkers across serial sections of a single tissue. These approaches will lead to the development of standardized quantitative paradigms with predictive power in evaluating patient drug response.

